# Advanced mycelium materials as potential self-growing biomedical scaffolds

**DOI:** 10.1038/s41598-021-91572-x

**Published:** 2021-06-16

**Authors:** Maria Elena Antinori, Marco Contardi, Giulia Suarato, Andrea Armirotti, Rosalia Bertorelli, Giorgio Mancini, Doriana Debellis, Athanassia Athanassiou

**Affiliations:** 1 Smart Materials, Fondazione Istituto Italiano Di Tecnologia, Via Morego 30, 16163 Genova, Italy; 2 DIBRIS, University of Genoa, Genoa, Italy; 3 Translational Pharmacology, Fondazione Istituto Italiano Di Tecnologia, Via Morego 30, 16163 Genova, Italy; 4 Analytical Chemistry Lab, Fondazione Istituto Italiano Di Tecnologia, Via Morego 30, 16163 Genova, Italy; 5 Electron Microscopy Facility, Fondazione Istituto Italiano Di Tecnologia, Via Morego 30, 16163 Genova, Italy

**Keywords:** Biomaterials, Fungi

## Abstract

Mycelia, the vegetative part of fungi, are emerging as the avant-garde generation of natural, sustainable, and biodegradable materials for a wide range of applications. They are constituted of a self-growing and interconnected fibrous network of elongated cells, and their chemical and physical properties can be adjusted depending on the conditions of growth and the substrate they are fed upon. So far, only extracts and derivatives from mycelia have been evaluated and tested for biomedical applications. In this study, the entire fibrous structures of mycelia of the edible fungi *Pleurotus ostreatus* and *Ganoderma lucidum* are presented as self-growing bio-composites that mimic the extracellular matrix of human body tissues, ideal as tissue engineering bio-scaffolds. To this purpose, the two mycelial strains are inactivated by autoclaving after growth, and their morphology, cell wall chemical composition, and hydrodynamical and mechanical features are studied. Finally, their biocompatibility and direct interaction with primary human dermal fibroblasts are investigated. The findings demonstrate the potentiality of mycelia as all-natural and low-cost bio-scaffolds, alternative to the tissue engineering systems currently in place.

## Introduction

**Figure 1:**
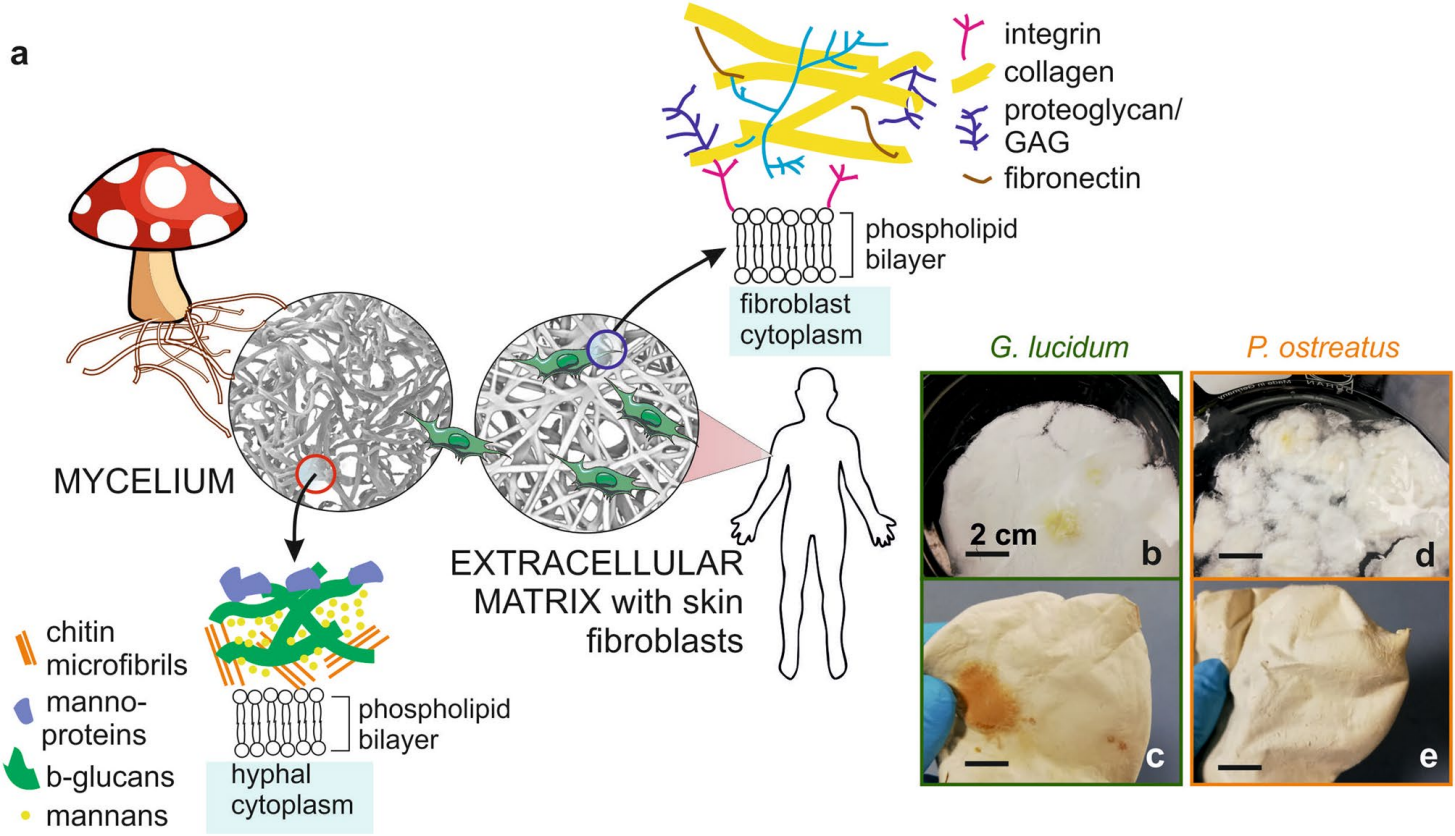
Mycelia as potential bio-scaffold materials. (**a**) Schematic representation of the microscopic structure and the cell composition of fungal mycelium and extracellular matrix, emphasizing their outer membrane components. (**b**–**d**) Photographs showing the macroscopic aspect of the two types of mycelia under study after their growth without further treatment (**b**–**d**), and (**c**–**e**) after autoclave.

Extracellular matrix (ECM) is composed of several macromolecules, and its role is to provide physical support, communication pathways, and 3D organization to the cells in organs and tissues (Fig. 1a)^1,2^. ECMs differ in terms of porosity, mechanical properties and biochemical cues for attachment, depending on the types of cells they support and the tissues in which they reside^3^. In the last decades, several efforts have been made in the field of tissue engineering to mimic this diversity and achieve specific tools to support the regeneration of human tissues. For this reason, various scaffold materials have been designed, and techniques such as electrospinning, freeze-drying and 3D printing have been developed and extensively employed. These top-down approaches allow a fine and accurate fabrication of suitable porous structures but require the use of solvents and sophisticated instrumentation^4–6^. Electrospinning generates aligned, micro- and nano-metric fibrous mats from many natural (*e.g.* silk^7,8^, keratin^9–11^, alginate^12,13^, chitosan^14^, collagen^15^) and synthetic (*e.g.* PLA^16^, PCL^17^, PVP^18^, and PEO^19^) polymers, by applying a high voltage to the initial polymeric solutions: although environmental-friendly solvents are being experimented, the use of toxic reagents might still be needed to achieve viscosity and concentration suitable for the fibers formation^20–22^. Similarly, viscoelastic properties constitute a key factor of 3D-printing inks, leading to the use of potentially harmful compounds, in addition to the employment of expensive 3D printers to ensure high resolution in the process^23,24^. On the other hand, water is the most predominant solvent in the freeze-drying technique, whose main drawback resides, however, in the use of expensive equipment to control the sublimation step^25^.

In combination with such technological advancements, the introduction of biopolymers ensured a big step forward in the production of functional and smart scaffolds. Biopolymers, such as PCL and PLA, succeeded in matching the requirements for biodegradable materials with prolonged life-time and mechanical resistance inside the body. On the other hand, the chemical moieties available in these synthetic polymers are not capable to actively promote cellular interactions^26,27^.

Polysaccharides and protein polymers, such as chitin^28^, alginate^29^, hyaluronic acid^30,31^, silk^32,33^, and keratin^9^ display biochemical guidance for cell migration, overcoming the lack of targeting for the attachment, typical of the aforementioned synthetic polymers^34^. Indeed, the hydroxyl groups present in polysaccharides and the fibronectin-specific amino acid sequences (RGD: Arginine–Glycine–Aspartic acid, and LDV: Leucine-Aspartic acid-Valine) displayed by protein-based biomaterials have proven to be extremely important for a successful cell adhesion^35,36^. Plant derived proteins and polysaccharides have proved to be more sustainable and less expensive than their animal counterpart, overcoming some issues in extraction and source recovery^37^. However, stable and water-resistant scaffolds can be obtained only after crosslinking or blending steps^38–40^.

Therefore, combining in one single material proper 3D-structure, suitable mechanical resistance, and bio-functional groups that can steadily and actively sustain the cell attachment still holds a great challenge. Recently, decellularization of animal and plant tissues has been proposed as a great solution to this issue, but several physical, chemical or enzymatic treatments are necessary preliminary steps to achieve an appropriated scaffold for cell growth and attachment^41,42^.

Mycelium, the vegetative part of fungi, is emerging as a highly tunable, self-growing, biodegradable, and low-cost biomaterial. Up to now, it has been exploited in an exploratory manner for packaging, textile and construction applications^43^. Mycelium is constituted of elongated cells, called hyphae, which expand uptaking nutrients from their growth substrate, spontaneously growing in fibrous mats. This is the case of the edible fungal species *Ganoderma lucidum* and *Pleurotus ostreatus* considered in this work^44^. Overall, mycelia present a self-grown porous structure and their hyphal outer membranes expose both polysaccharides and proteins (Fig. 1a). Furthermore, in our previous works, it has been reported that morphology, chemical composition, hydrodynamics and mechanical properties of mycelia can be finely tuned by changing both the fungal strain and their growth substrate^45–47^.

The scientific works reported so far on the use of mycelia for biomedical applications or on the investigation of their biocompatibility concern their derivatives. Indeed, several fungal strains are reported to secrete bioactive polysaccharides, mainly chitin-derivatives, that can be extracted either from the growth medium or directly from the mycelial network using strong bases or acids^48^. Membranes produced by simply casting these extracts, such as the Saccachitin reported by Su et al.^49^ and the Rhizochitin produced by Chien et al*.*^50^, showed positive effects on fibroblast and keratinocytes growth. As far as we know, only the work by Narayanan et al*.* reported the use of *Aspergillus* sp. as scaffolds for the culture of human keratinocytes, but such application was possible only after a strong chemical treatment of the mycelia with β-mercaptoethanol^51^. Here, for the first time, we seek to demonstrate how the entire fungal mycelial structure can be directly employed, without chemical pretreatments, as a valid and sustainable alternative for the controlled fabrication of all-natural, self-grown and low-cost biomedical scaffolds. Thus, we present the growth and characterization of *Ganoderma lucidum* and *Pleurotus ostreatus* mycelia as tissue engineering adhesion platforms, and we report preliminary, yet promising results onto their biocompatibility towards a primary human cellular model.

## Materials and methods

### Strain, media and growth conditions

*Ganoderma lucidum* DSM9621 and *Pleurotus ostreatus* DSM11191 active cultures were purchased from DSMZ (Germany) and maintained in 100 mm Petri dish with Potato Dextrose Broth (PDB, Merck) as growth medium, transferring the culture to fresh medium every 30 days. A piece of 20 day-grown mycelium was inoculated in 100 mm Petri dishes containing 30 mL of PDB at 24 g/L in water. Media were autoclaved before use, at 120 °C for 20 min by SYSTEC-VX 40. Mycelia were incubated in a climatic chamber (Memmert, HPP 260) at 27 °C and 78% relative humidity, in the dark.

### Material preparation

After 20 days of growth, when the whole surface of the plate was covered, mycelium was collected, cleaned from the substrate with a spatula and deionized water. Mycelia were then either dried for 15 h at 50 °C in an oven or autoclaved at 120 °C for 20 min. Autoclaved mycelia were then dried under a laminar fume hood and illuminated for approximately 100 min with UV light.

### SEM and TEM analyses

Mycelia were fixed in a solution of 2% glutaraldehyde in 0.1 M cacodylate buffer for 2 h at room temperature. After several washes in the same buffer, the samples were post-fixed in 1% osmium tetroxide in MQ water for 2 h and washed with MQ water. Mycelia were subsequently dehydrated with a series of 10-min incubations in rising concentrations of ethanol in water solutions (from 30 to 100%), 1:1 ethanol: hexamethyldisilazane (HMDS, Sigma-Aldrich) and 100% HMDS and dried overnight in air. Finally, the samples were sputtered with a 10 nm gold layer and analysed using a JEOL JSM-6490LA Scanning Electron Microscope (SEM) equipped with a tungsten filament and operating at 10 kV of accelerating voltage.

For TEM analysis, after fixation with glutaraldehyde as the previous description, mycelia were post-fixed in 1% osmium tetroxide in MQ water for 2 h, washed in MQ water and stained overnight at 4 °C in an aqueous 0.5% uranyl acetate solution. Then, the samples were dehydrated in a graded ethanol series and embedded in SPURR resin for 48 h at 70 °C. Sections of about 70 nm^52^ were cut with a diamond knife on a Leica EM UC6 ultramicrotome. Transmission electron microscopy (TEM) images were collected with a Jeol JEM 1011 (Jeol, Japan) electron microscope equipped with a 2 Mp charge-coupled device camera (Gatan Orius).

### Density and porosity

Skeletal density was measured by helium pycnometry Thermoscientific Pycnomatic Evo with a 44 cm^3^ chamber. Measurements were performed at 20 °C. The real (or skeletal) density is the sample mass referred to the sample volume, excluding all pores and void volumes but considering “closed” pores (*i.e.* cavities within the material that cannot be reached by any gas). Skeletal density was measured by detecting the change in pressure due to the volume of helium that is displaced by the sample within the sealed and pressure-equilibrated chamber. Helium is a tiny atom that can permeate even extremely narrow pores in a solid, thus permitting the determination of the real volume occupied by that solid. The ratio of the dried mass and its volume gives the real density of the material under test as a result. Ten measurements were averaged for each sample. Porosity was determined by mercury intrusion porosimetry (MIP) performed with Pascal 140 Evo and Pascal 240 Evo mercury porosimeters (Thermo Scientific). In this technique, the sample is entirely embedded by mercury. The pressure is then increased so that mercury starts entering in the pores. The total porosity (estimated by MIP) is related to the volume of mercury totally intruded at the end of the measure. The porosity, in fact, is expressed as the ratio between the pore volume (inner cavities volume) and the external sample volume, while the pore size distribution depends on the volume of mercury intruded at each pressure range. The pressure of mercury intrusion was set at 0.0136 MPa and continuously increased up to 200 MPa, with a rate of 6–14 MPa min^−1^. The contact angle of mercury with the samples and the surface tension of pure mercury were assumed to be 140° and 0.48 N m^−1^, respectively. Washburn equation was used to calculate the pore size from the applied pressure, assuming that the pores are of cylindrical shape^45^.

### Chemical analysis

Infrared spectra of samples were obtained with an attenuated total reflection (ATR) accessory (MIRacle ATR, PIKE Technologies) coupled to a Fourier transform infrared spectrophotometer FTIR spectrometer (Vertex 70v FT-IR, Bruker). All spectra were recorded in the range from 3800 to 600 cm^−1^, with 4 cm^−1^ resolution, accumulating 64 scans. The sample was gently placed on a spot of ATR accessory and slowly pressed, with the part grown in contact with the substrate (named “bottom”) on the ATR crystal. To ensure the reproducibility of the obtained spectra, three samples of each type are measured^45^. Spectra analysis was performed with Origin pro 2016 software.

### Hydrodynamic characterization

A contact angle (CA) goniometer (DataPhysics OCAH 200) was used for static water contact angle measurements at room temperature. Five μL droplets of water were deposited on the corresponding surfaces and side-view images of the drops were captured after 60 s. CA were automatically calculated by fitting the captured drop shape. Up to 15 contact angle measurements were carried out on every sample at random locations and their average values and standard deviation were reported^45^. Contact angle was measured after a conditioning in either a dry or a humid environment (*i.e.* conditioned for 24 h at 100% RH). For the water uptake, dry samples were weighed on a sensitive electronic balance and then placed in different humidity chambers. Samples were dried by conditioning in a desiccator for 24 h, weighed, and then transferred in 100% humidity conditions for additional 24 h, before being weighed further. The amount of adsorbed water was calculated based on the initial dry weight.

### Mechanical characterization

Samples were cut into 20 × 35 mm^2^ rectangles and tested at room temperature, after 24 h conditioning at 100% RH. Tensile stress curves were obtained by a dual column universal testing machine (Instron 3365): samples were mounted on the machine clamps and deformed at a rate of 1 mm/min until failure. The Young’s modulus E, ultimate tensile strength UTS and elongation at fracture were extracted from the stress–strain curves^45^. Storage modulus E’ and tanδ (*i.e.* the ratio between loss and storage moduli, representing the relative energy dissipation) were also measured with a Q800 DMA testing machine (TA Instruments), in uniaxial tensile mode, applying a sinusoidal deformation with an amplitude of 20 µm, and frequencies of 7, 10 and 16 Hz^45^.

### Mycelia extract characterization

#### In vitro biocompatibility

Primary human dermal fibroblasts (HDFa, Thermo Fisher Scientific) were used as a cellular model to investigate the biocompatibility of the grown mycelia. After being cultured in T75 flasks in the presence of Medium 106 supplemented with LSGS Kit (Thermo Fisher Scientific), cells were seeded onto 24-well plates at a density of 7000 cells/cm^2^ and let attach overnight in an incubator at 37 °C and with 5% CO_2_. *P. ostreatus* and *G. lucidum* extracts were prepared as following. Autoclaved mycelia were cut in pieces of about 20 mg and sterilized under the UV light for 20 min (10 min per side). To remove the excess of PDB from the fungi matrices, the pieces were immersed in sterile potassium phosphate buffer (PBS, pH 7.4, Gibco) and incubated at 37 °C for 24 h. A second washing was performed with fresh PBS for additional 24 h. Afterwards, each 20-mg mycelia piece was incubated with 1 mL of Medium 106 for the following 24 h and the resulting stock solutions were used to prepare the tested dilutions (1:2, 1:3 1:4 1:20, 1:40, 1:100). Attached HFDa cells (at passages 4–6) were treated with mycelia extracts for 24, 48, and 72 h, while cells incubated in normal Medium 106 + LSGS were considered as controls. MTS assay (CellTiter 96^®^ AQ_ueous_ One Solution Cell Proliferation Assay, Promega) was conducted to determine cell viability. Briefly, all samples were incubated in fresh culture media (500 µL) and 25 µL of reagent were added to each well. After 3.5 h of incubation, absorbance readings at 490 nm were recorded. Three independent experiments were carried out in triplicates. A Student’s *t*-test, assuming unequal variances, was carried out, considering a *p* < 0.01 value.

To further assess the biocompatibility of the mycelia matrices, a semi-contact assay was performed. Primary human fibroblasts (at passages 4–6) were seeded onto 24-well plates at density of 7000 cells/cm^2^ and let attach overnight. *P. ostreatus* and *G. lucidum* were cut in small pieces (with weights ranging from 1 to 5 mg) and washed and sterilized as previously described. The next morning, fresh culture medium (1 mL) was replenished and a piece of mycelium was gently immersed in each well and let float for additional 24, 48, and 72 h. Afterwards, the matrices were carefully removed, paying attention not to perturb the layer of attached cells at the bottom of the well, and the MTS assay was carried out. Three independent experiments were carried out in triplicates. A Student’s *t*-test, assuming unequal variances, was carried out, considering a *p* < 0.01 value.

In order to visualize the morphology of the fibroblasts subjected to the various treatments, cells were plated onto glass coverslips at a density of 5000 cells/cm^2^ and treated as described above. Afterwards, cells were washed with pre-warmed PBS and fixed with 3.7% paraformaldehyde for 20 min. A DAPI solution (2.5 μg/mL) was used to stain the cell nuclei (15 min in the dark). To allow the actin fibers staining, fibroblasts were permeabilized with 0.3% Triton X-100 (8 min), prior to incubation in Alexa Fluor 488 Phalloidin (Thermo Fisher Scientific, 1:100 dilutions in PBS) for 20 min in the dark. The prepared coverslips were then mounted onto glass slides with Fluoromont-G and imaged with a confocal microscope Nikon A1.

#### Cell plating onto the *P. ostreatus* matrices

With the aim of investigating the mycelia suitability as substrates for cell attachment and growth, human primary fibroblasts were used as a challenging platform. Briefly, mycelia matrices were cut into round pieces (1 cm in diameter) and sterilized/washed, as reported in the previous paragraph. The substrates were placed at the bottom of a 24-well plate and kept firm with sterile PDMS rings (outer diameter = 1.5 cm, inner diameter = 0.8 cm). Some of the matrices were incubated with 400 µL of fibronectin (20 µg/mL, Fibronectin Human Protein, Thermo Fisher Scientific) for 1 h at 37 °C, while other matrices were left uncoated and incubated in sterile PBS. After fibronectin/PBS removal, the matrices were dried for 2 h under a sterile hood and seeded with cells at a density of 5000 cells/cm^2^. After 48 h of culture, samples were fixed in 3.7% paraformaldehyde. In order to partially block the autofluorescence signal of the fungal substrate, all the staining solutions (DAPI and Alexa Fluor 546 Phalloidin) were prepared in 1% Bovine Serum Albumin and the samples were processed as described above. The stained and glass-mounted substrates were imaged with a confocal microscope Nikon A1, equipped with a 560 nm laser. Images were acquired with both PMT and spectral detector (spectral acquisition from 541 to 679 nm, grating resolution of 6 nm). The image analysis (spectral unmixing, ROI definition and spectral profiling) was carried out with ImageJ (https://imagej.nih.gov).

#### SEM imaging of the *P. ostreatus* cell scaffolds

*P. ostreatus* matrices, prepared as above-mentioned and seeded with HDFa cells for 48 h, were treated as reported above and observed by JEOL JSM-6490LA Scanning Electron Microscope equipped with a tungsten filament and operating at 10 kV of accelerating voltage.

#### High resolution UPLC-mass spectrometry

The samples were dried under nitrogen and then reconstituted in 10% acetonitrile in water. Five microliters of these samples were then injected in an Acquity UPLC liquid chromatography system coupled with a Synapt G2 QToF high-resolution mass spectrometers (both from Waters, Milford, MA, USA). The analytes were then separated on a BEH (2.1 × 100 mm) reversed-phase column (Waters) using a linear gradient of acetonitrile in water (5 to 100%). The eluting compounds were analyzed by high-resolution mass spectrometry in both positive and negative ion electrospray modes. Leucine Enkephalin reference standard was used as lock-mass to achieve a mass accuracy below 5 ppm. Metabolites were tentatively identified by interrogating the publicly available HMDB (Human Metabolome Database) and LipidMaps reference databases.

## Results and discussion

### Growth, harvesting and inactivation of mycelia

**Figure 2:**
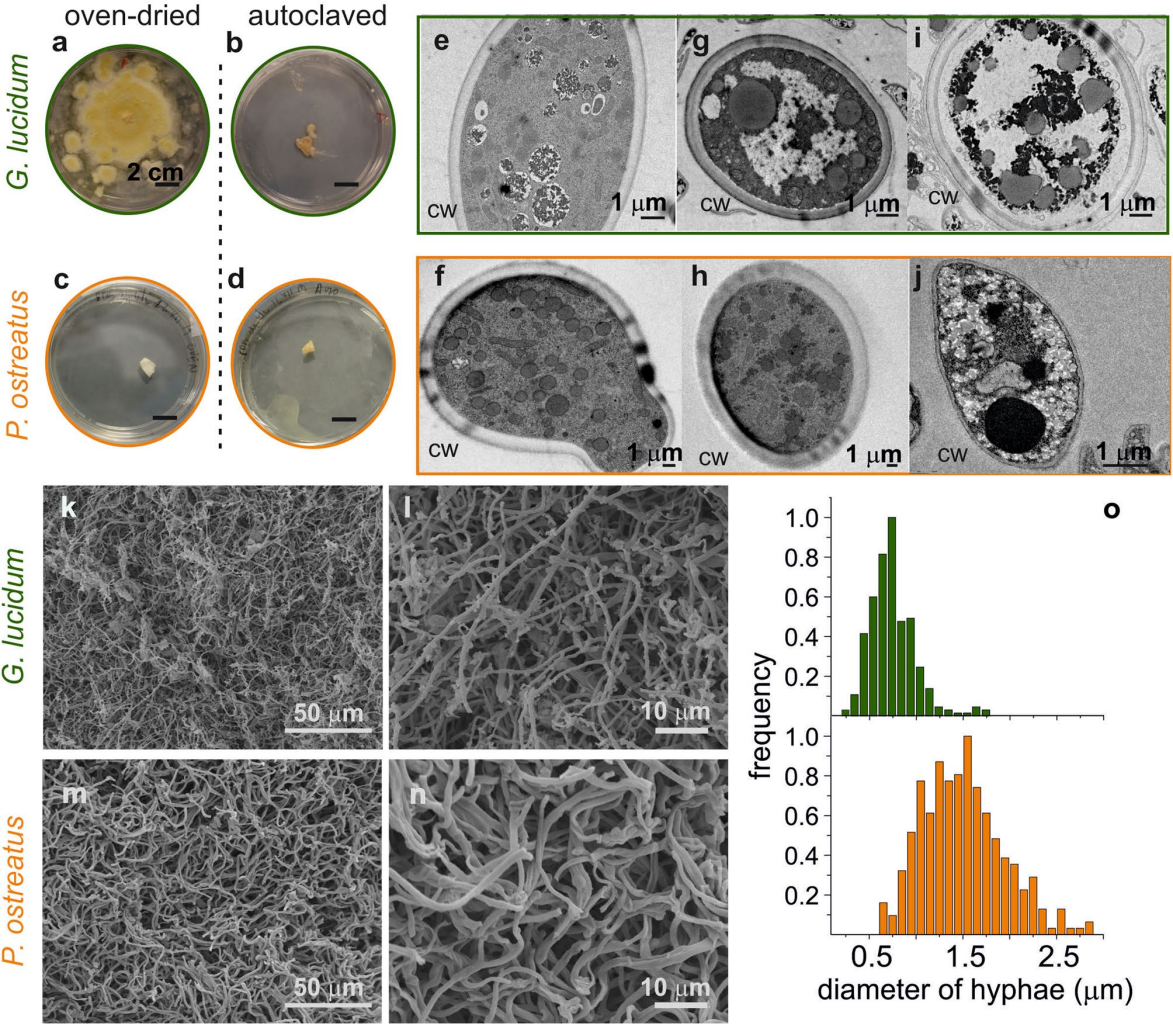
Morphological features of mycelia. (**a**–**d**) “Re-growth” experiments with oven-dried and autoclaved mycelia (**e**–**j**) TEM images of mycelia as grown (**e**, **f**), dried in oven (**g**, **h**) and autoclaved (**i**, **j**), *CW* = *cell wall*. (**k**, **n**) SEM images of autoclaved mycelia; (**o**) distribution of hyphal diameters as calculated from the SEM images of the autoclaved mycelia.

The fungal mycelia were grown onto Petri dishes filled with potato dextrose broth (PDB) inside a climatic chamber (27 °C, 78% R.H.) until complete surface coverage, to be then collected and cleaned from the substrate (Fig. 1b–-e). After the harvest, the complete inactivation of the biological activity of the mycelium, to halt its growth, constituted the first crucial step before testing it as a potential biomaterial scaffold. Mycelium inactivation is usually performed via heat treatment^53^. However, in a control experiment in which pieces of oven-dried mycelia (50 °C for 15 h) were placed back in contact with PDB, *Ganoderma* occasionally re-grew, unlike *Pleurotus* (Fig. 2a,c), suggesting that this weak treatment could be insufficient to achieve a stable configuration. This phenomenon was not completely unexpected since the heat-resistance of growing strains of *Ganoderma lucidum* has already been observed^54^, contrarily to *Pleurotus ostreatus*^55^. Therefore, the autoclave was chosen as post-growth treatment, since both strains appear inactive after being subjected to it (Fig. 2b,d). Transmission microscopy (TEM) investigation showed that after the oven treatment (Fig. 2g,h), the inner cell organization was comparable to the control (Fig. 2e,f). Instead, after autoclaving (Fig. 2i,j), the hyphal cells content drastically changed: large white areas appeared inside the cells, probably due to plasmolysis, and vacuoles formed as a consequence of the heat and pressure shocks^56^. Importantly, the main structural component, the cell wall (CW), was still intact in both mycelia, even if it slightly shrunk in *Pleurotus*. The cell diameter was comparable between oven-dried and autoclaved mycelia, suggesting that cell shrinkage, usually reported for fungi undergone strong thermal treatment, does not occur in this case^57^. Given these results, further analyses were only performed onto autoclaved samples.

### Morphological characterization

Scanning electron microscopy (SEM) investigation of post-autoclave mycelia allowed the observation of the hyphal structure and the calculation of their diameters (Fig. 2k–n), both essential features for a biomedical scaffold. Fibers were randomly oriented, and the strains presented the same morphological differences already observed by Haneef and colleagues with oven dried mycelia^46^. In *Ganoderma lucidum* two kinds of hyphal structures were noticeable, a *tube-like* short one, and a long smoother one, defined as *thread-like*. Instead, *Pleurotus ostreatus* mycelia were only composed by the latter kind. Distribution of hyphal diameters was comparable to what previously reported for oven-dried mycelia: *Pleurotus* hyphae are larger, measuring on average 1.5 ± 0.4 μm, while *Ganoderma* thread-like ones average at 0.7 ± 0.2 μm (Fig. 2o). Such fibrous network arrangements and dimensions have been reported to be suitable for cell attachment^58–60^.

**Figure 3:**
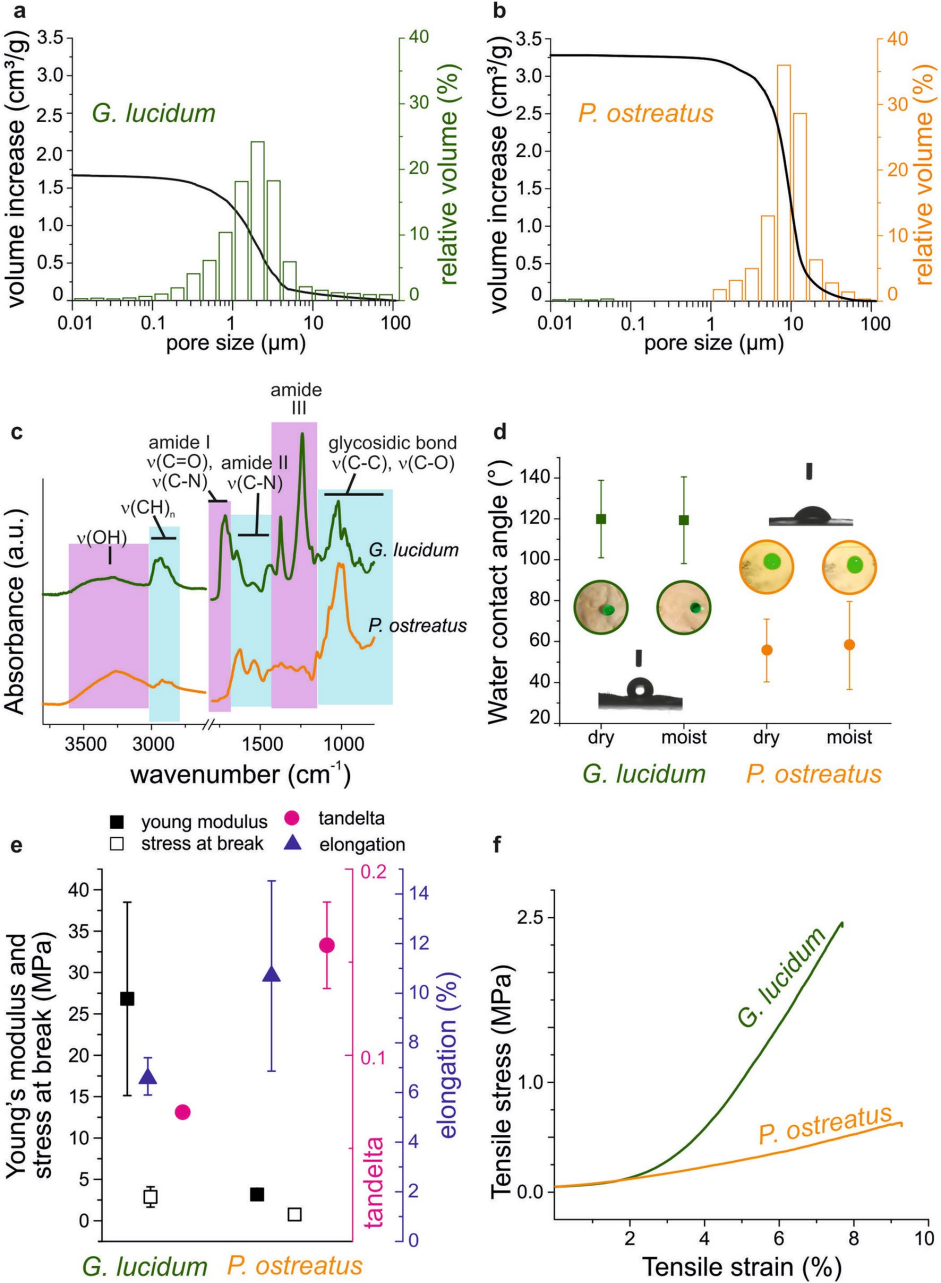
Porosity, chemical, hydrodynamical and mechanical features of mycelia. (**a**, **b**) Pore diameter distribution (bars) and total porosity (curves) measured by MIP; (**c**) ATR-FTIR spectra of the two mycelia; (**d**) Contact angle of dry and moist mycelia. Inset: colored water drops on mycelium pieces of both strains, either dry or moist. The drops volume is 5 μL. (**e**) Young modulus, stress at break and elongation results after the tensile test; tanδ values after DMA analysis. (**f**) Tensile test curves of the two mycelia under study.

Being able to greatly affect cell migration, oxygen regulation and nutrient exchange, porosity is a key parameter that should be considered during a scaffold design^61^. Usually, in tissue engineering applications, average pore size is measured by the post-processing of SEM micrographs, while total porosity can be calculated separately by liquid displacement method, typically employing ethanol^47,62,63^. In this work, both total porosity and size pore distribution were inferred from mercury intrusion porosimetry (MIP, see Experimental Methods section) analysis, to ensure that the whole range of sizes presented by these natural, highly-variable materials, could be included (Fig. 3a–-b). In particular, the determination of total porosity depends on the whole amount of mercury volume intruded (black curve in Fig. 3a, b), while information on different pore sizes is calculated from the amount of volume intruded at each pressure (bars in Fig. 3a, b). To avoid false positives given by pore opening under the high pressures characteristic of MIP, the analysis was preceded by skeletal density measurements by helium picnometry^64^. Skeletal density is the ratio between the dry weight of the sample and the volume of the sample, excluding all the open pores. Being a tiny atom, helium succeeds in measuring even smaller pores, with fewer risks of artefacts in soft materials, such as mycelia^65,66^.

*Pleurotus* 3D network resulted denser (1.48 ± 0.03 g/cm^3^) than *Ganoderma* (1.34 ± 0.02 g/cm^3^; data not shown), and it was also characterized by a higher over-all porosity (85%) than that of its counterpart (68%). The pore size distribution showed that the majority of mercury was intruded in pores between 1 and 5 µm for *Ganoderma*, while in holes between 7 and 20 µm for *Pleurotus*. Overall, these findings suggest the potential ability of different mycelial networks to host specific cell types or cellular clusters, depending on their hyphal architecture and overall porosity. In particular, considering the scope of this preliminary investigation, fibroblasts show to better infiltrate scaffolds presenting pores from 20 to 100 µm, but smaller pores are reported to favor cells adhesion, bridging and ECM production^60,67,68^.

### Chemical composition of mycelia

The presence of a wide variety of chemical functionalities on their surface makes mycelia an interesting platform for tissue engineering applications. Cells preferably attach onto carbohydrate and protein moieties, namely by interacting with the numerous proteoglycans and proteins present in the extracellular matrix (ECM). In particular, carboxyl, sulfate and hydroxyl groups exhibited by glycosaminoglycans and the aminoacidic RGD motifs presented on ECM proteins (*i.e.* collagen and fibronectin) are the main responsible for cell attachment^69^. Usually, polymers and biomaterials employed for scaffold biofabrication do not naturally display all the above-mentioned chemical functionalities, which must be added to the scaffold surface in a second step. For example, cellulose can be phosphorylated to improve its biocompatibility, while polymeric electrospun fibers can be enriched by sulfated compounds^70^. ATR-FTIR analysis (Fig. 3c) of the self-grown materials reveals how hydroxyl, carboxyl and amide groups are present in both strains since polysaccharides, lipids and proteins compose the fungal cell wall (Fig. 1a)^71–73^. The main differences in the ATR-FTIR spectra between the two strains under study concern the area corresponding to the amide bond stretching. *Ganoderma* mycelium shows one peak at 1715 cm^−1^ (amide I stretching area) and two peaks at 1375 and 1240 cm^−1^ (amide III stretching range), which are not visible in the *Pleurotus* spectrum. On the other hand, this latter presents two peaks at 1620 and 1540 cm^−1^ (amide II stretching range), differently from the *Ganoderma* spectrum, where only one peak at 1450 cm^−1^ is visible in the same region. Differences in these ranges can be related to variations in chitin content, which broadly influences the mycelial properties (*i.e.* hydrophobicity and mechanical resistance)^46^. As previously mentioned, cells preferably attach onto carbohydrate and protein moieties^69^. Therefore, the observed wide variety of chemical functionalities on the fungal surface indicates a promising path towards the development of fully fungal-based biomaterials for tissue engineering.

### Hydrodynamic characterization

The hydrodynamic behavior of the developed, autoclaved, mycelia was characterized through the static water contact angle (CA) and the ability to adsorb water moisture in a saturated water atmosphere. Contact angle was measured after conditioning for 24 h the mycelium substrates both in a dry and a humid environment, since applications in a water-containing medium are being considered in the second part of this work (Fig. 3d). *Ganoderma* displayed a stable CA of ≈119° under both conditions. At the same time, *Ganoderma* mycelia present a limited capability of moisture adsorption, since only a 30 ± 1% weight increase was recorded after placing the material in a humid chamber (data not shown). On the other hand, *Pleurotus* mycelia exhibited lower CA values in both conditions, *i.e.* CA was 56 ± 15° for dry mycelia and 58 ± 21° for moist ones. These mycelia were more prone to adsorb moisture, with values reaching up to an increase of 68 ± 6% of their weight after 24 h in a humid chamber.

The differences in chemical composition and porosity reported above can explain the diverse hydrodynamic behavior, especially in terms of moisture uptake. Moreover, the resulting water contact angle strongly depends on the entire surface topography^74,75^. Generally, more hydrophilic surfaces are thought to favor cell attachment^76^, but studies have shown that protein can adhere even to more hydrophobic surfaces^77^, especially after increasing the number of seeded cells or culture time^78–80^. Therefore, the diversity in hydrodynamic properties does not exclude any of the mycelia from further testing of biocompatibility and suitability to cell attachment.

### Mechanical characterization

Within the human body, tissues stiffness is in the range of kPa–MPa, and cells differentiate, grow and respond to their surroundings depending on the mechanical properties of the substrate^81^. Mycelia, in their dry state, are brittle materials, a characteristic that requires their combination in composites for constructions, thermal/acoustic isolation, and design manufacturing^82–84^. Consequently, obtaining precise measurements of mycelial mechanical properties can be challenging, given their inherent fibrous nature^85^. Therefore, the mechanical properties investigation was herein conducted both via tensile tests and via Dynamic Mechanical Analysis (DMA, Fig. 3e, f). Mycelia were conditioned in a humid atmosphere (100% RH) before the measurements, as they are here envisioned for wet applications. *Ganoderma lucidum* showed a higher Young’s modulus of 26.8 ± 11.7 MPa and a smaller elongation of 6.6 ± 0.8%, with respect to the *Pleurotus ostreatus,* that presented a Young’s modulus of 3.2 ± 0.1 MPa and an elongation of 10.7 ± 3.8%. Instead, the recorded stresses at break were only slightly different, *i.e.* 2.9 ± 1.2 MPa for *Ganoderma* and 0.7 ± 0.3 MPa for *Pleurotus*. Considering these results and the calculated tan-delta values, *Ganoderma* mycelium appears stiffer, while *Pleurotus* results more ductile. Overall, the stiffness of mycelia is comparable to values reported for other materials employed in tissue engineering, especially for skin mimicking^60,86^. In particular, mechanical properties of mycelia closely resemble those measured in living-tissues on a micrometric scale, favoring in vitro cell adhesion and tissue formation^87,88^.

### Primary human dermal fibroblasts response to fungal mycelia

#### Biocompatibility assays

A number of derivatives from fungal materials have been previously tested for their ability to sustain cell growth. Some fungal strains (*e.g. Ganoderma, Fusarium, Aspergillus*)^89^ are reported to secrete bioactive polysaccharides (usually branched heteroglucans or glycoproteins) that can be obtained from the growth medium^48,90–92^. On the other hand, the direct use of strong bases or acids onto the mycelia matrix, followed by casting of the extracted molecules, allows the production of mycelium-derivatives, which can work as substrate for cell culture. Examples are the Saccachitin reported by Su and colleagues and the Rhizochitin produced by Chien et al*.*, where the chitin extracted from mycelia is casted in membranes tested as skin substitute and for the growth of fibroblasts^49,50^ or for its immunomodulatory activity^93^.

In light of the promising results obtained by the above-mentioned groups, the herein project took a step forward, considering the entire mycelia as a potential scaffold for tissue growth and restoration. Hence, both the potential therapeutic effect and the three-dimensional, ECM-like structure of the mycelia would be preserved, by a procedure which does not require the use of any solvent nor any mechanical treatment, differently from what was reported by Narayan et al*.* for *Aspergillus* sp. Mycelia^51^. In this work, the only required step after collection of the mycelia is autoclave.

**Figure 4:**
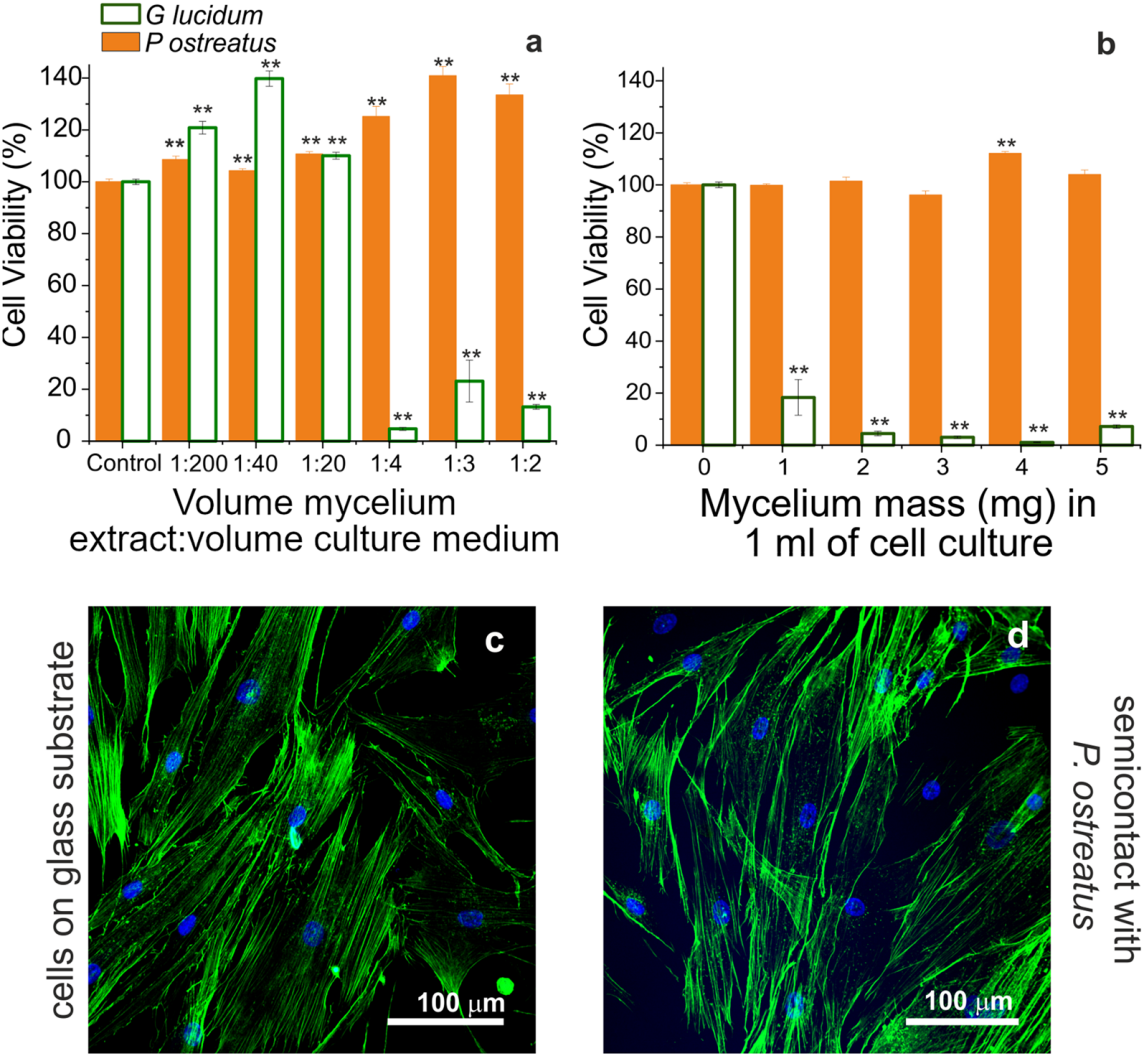
Biocompatibility towards HDFA cells. (**a, b**). Primary human fibroblast (HDFa cells) viability in the presence of mycelia extract (**a**) or semi-contact (**b**) as measured by MTS assay after 24 h. The reported values indicate the dilutions prepared starting from a stock solution of 20 mg of mycelium in 1 mL of Medium 106 containing growth factors. Data are expressed as average ± standard error and a *p* < 0.01 (**) was considered as significant. (**c, d**). Confocal images of control cells plated onto glass substrates and treated with Medium 106 containing growth factors (**c**), or in semi-contact with a 5 mg-piece of *P. ostreatus* (**d**).

A preliminary biocompatibility investigation was conducted following the ISO10993-5 standard test, which assessed the cyto-toxicity effects of a cell culture medium extract of the whole mycelium materials. Experiments were performed with primary human dermal fibroblast adult cells (HDFa)^10^. The viability of cells grown in the presence of the mycelium cell culture medium extracts was first assessed by MTS assay (Fig. 4a and Figure S1a,b ). In addition, a “semi-contact” experiment was carried out (Fig. 4b, and Figure S1c,d), where fibroblasts attached to the bottom of 24-wells plates were cultured in the presence of a piece of mycelium kept floating on top of them. From the experiments performed with *Pleurotus ostreatus*, a maximum viability was recorded for each condition tested (Fig. 4a, b, full orange bars). Moreover, these fibroblasts displayed the same morphology of the control cells (supplemented with culture medium), as noticeable via actin staining and confocal microscopy (Fig. 4c, d). Thus, mycelium from *Pleurotus ostreatus* appeared a promising substratum for tissue engineering applications. On the contrary, the preliminary assays conducted in the presence of *Ganoderma lucidum* revealed a different trend: in the “extract” experiments, the cyto-compatibility slightly increased with respect to the control samples for high dilutions (1:200 and 1:40 mycelia extract/culture medium, v/v) and then remarkably dropped in the presence of more concentrated extracts (reaching a viability of 13.2 ± 0.9% when diluting 1:2, Fig. 4a, empty green bars). Likewise, when the extract treatment was carried out for longer time points, only the high dilutions of the extracts (from 1:200 to 1:20) were not affecting the cell viability (Figure S1a,b). This might suggest a slight stimulating effect at low *Ganoderma lucidum* concentrations, which would require further investigations.

However, when pieces of *Ganoderma lucidum* were directly placed in the culture wells for 24 h, fibroblasts did not survive. To gain some insights on a possible explanation for this outcome, extracts from the two strains were analyzed by high-resolution UPLC-MS (ultra-performance liquid chromatography – mass spectrometry). This experiment revealed a significant amount of a *Ganoderma* metabolite even after two 24 h-washing steps with sterile PBS. This metabolite (Ganoderic acid V, a highly oxygenated lanostane-type triterpenoid, Figure S2) is known to have apoptotic and cytotoxic activities^94,95^. The toxic glucoside oleandrin was also detected in the extract, probably causing the fibroblasts death^96^. On the other hand, the same analysis on *Pleorotus* showed that hydroxylated fatty acids are the major metabolites (Figure S3), which resulted harmless for the primary cells.

#### Direct growth of primary human fibroblasts on *Pleurotus ostreatus* mycelia

**Figure 5:**
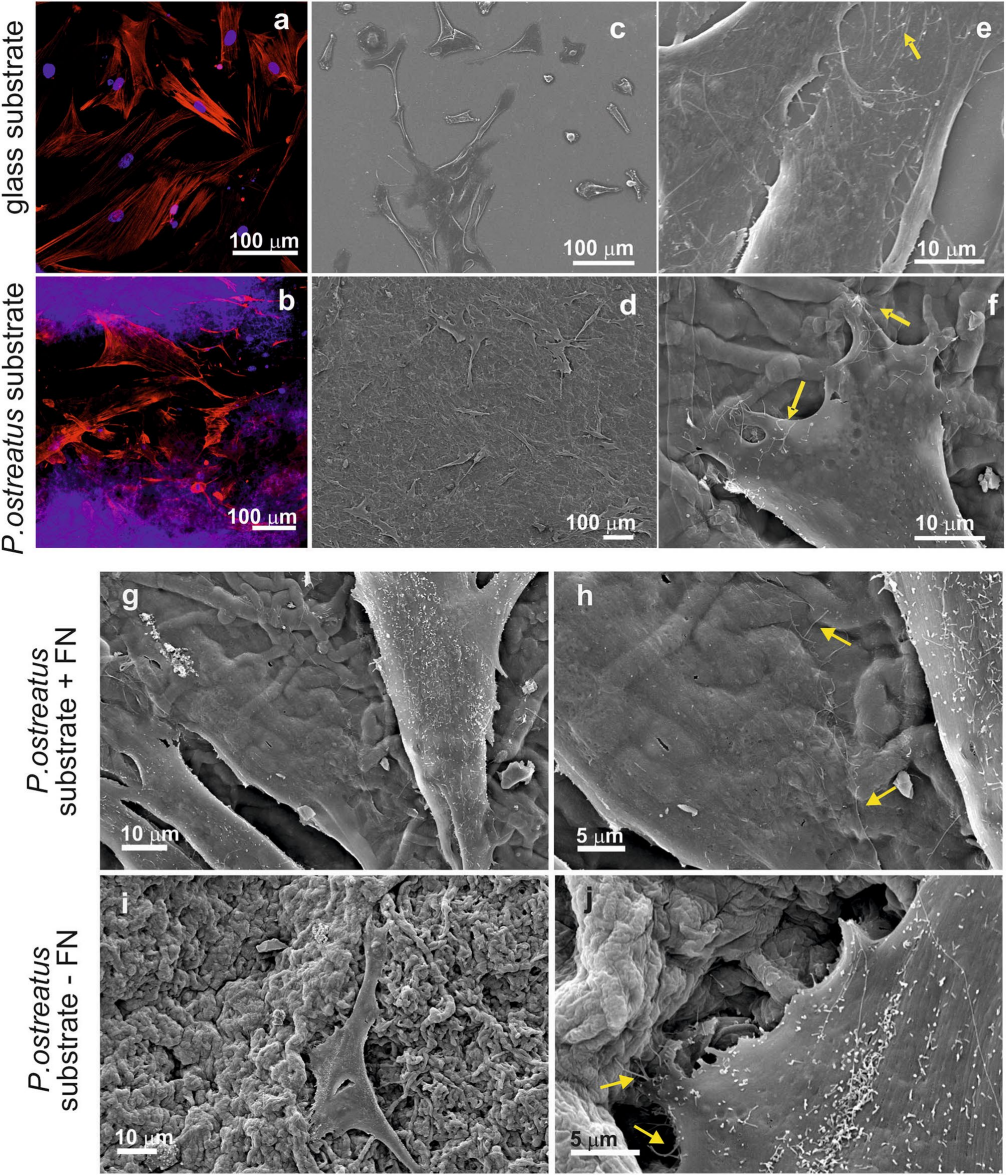
Confocal (**a**) and SEM micrographs (**c**, **e**) of HDFa cells grown for 48 h onto a FN-coated glass coverslips. Confocal (**b**) and SEM micrographs (**d**, **f**) of HDFa cells grown for 48 h onto a FN-coated *P. ostreatus* substrate. Nuclei are stained with DAPI (visible in blue), while the actin fibers are stained with Alexa Fluor Phalloidin 488 (highlighted in green). Filopodia extending from the cytoplasm of the attached cells are indicated with yellow arrows. (**g**–**j**) Effect of the scaffold coating onto primary cell growth. HDFa cells are seeded onto *P. ostreatus* scaffolds, either coated with fibronectin (**g**, **h**) or uncoated (**i**, **j**). Filopodia extending out of the attached cells are highlighted with yellow arrows.

Considering the preliminary biocompatibility outcomes, primary fibroblasts attachment was tested only onto the mycelia from *Pleurotus ostreatus*. Experiments were conducted either onto Fibronectin (FN) coated substrates (Fig. 5d, f, g, h) or onto FN-uncoated mycelia (Fig. 5i, j), to observe if the presence of this ECM protein known to mediate cell-substrate interaction could be necessary to achieve cell adhesion. As observed by confocal (Fig. 5b) and SEM microscopy, cell attachment was successful, with primary fibroblasts adhering to the corrugated, wavy, 3D mycelium matrix, and presenting a healthy morphology (comparable to that observed on FN-coated control glass slides, Fig. 5a, c, e). Interestingly, the SEM investigation revealed the presence of cytoplasmic filaments (filopodia, indicated by yellow arrows in Fig. 5f, h, j) protruding from the leading edges of the cells. This phenomenon is more evident for the cells plated on the *Pleurotus ostreatus* scaffolds, either FN-coated or non-functionalized, as the filaments appeared anchored to the hyphae. As expected, a denser sheet of adherent fibroblasts was observed with the FN surface functionalization, indicating that the ECM protein coating is useful but not essential to promote primary fibroblasts attachment onto *Pleurotus ostreatus* substrates^97,98^.

Further investigations will be carried out, to study the distribution of the cellular focal adhesion complexes with respect to the mycelia hyphal structure, and to investigate if mycelium-based materials can also actively promote fibroblasts growth and migration^99^. Deeper insights into the mechanisms of cell attachment onto the mycelial surface might arise from studies employing high-resolution microscopy and molecular biology tools.

## Conclusions

In conclusion, mycelia of filamentous, not pathogenic fungi having a spontaneously-formed tridimensional biopolymeric network constitute ideal self-growing, all-natural biocomposite scaffolds for cellular growth. We demonstrated for the first time that mycelium from *Pleurotus ostreatus* can be directly used as scaffold for the growth of cells, determining attachment of primary human fibroblasts with excellent viability and morphology comparable with that observed in the control samples. A simple and fast autoclaving process was the only treatment performed onto the self-grown composite biomaterial, serving the double purpose of completely inactivate the fungal spores and sterilize the scaffold via a standardized method. To the best of our knowledge, this is the first report of primary human cells directly plated onto an un-modified and non-functionalized mycelial scaffold. Results obtained with another filamentous fungus, *Ganoderma lucidum,* showed that complete inactivation of the hyphal cells is necessary but not enough to grant biocompatibility, as its water extract contained picomolar concentrations of organic acids (Ganoderic acid V and Oleandrin), which resulted detrimental for the HDFa cells survival.

Overall, the physico-chemical features (*i.e.* morphology, chemical composition and wettability) of the herein presented self-grown biomaterials suggest the great intrinsic potentialities of mycelia as tunable platforms for tissue engineering. Exploiting the different properties combinations resulting from the coupling between a specific fungal strain and its growth substrate can pave the way to a new generation of scaffold design. Mycelium can be proposed as a novel, more sustainable, “bottom-up” approach for the fabrication of a simulated ECM, advanced alternative to other top down and bottom up systems currently used as scaffolds for tissue engineering. We forth see a broad variety of functional, biocomposite mycelium-based constructs that can be designed and exploited for numerous applications in the biomedical field, leading to a transformation in the way the biomaterials are conceived and fabricated.

## Supplementary Information

Supplementary Information.
