# Supplementary material for: Advanced mycelium materials as potential self-growing biomedical scaffolds
Source: Sci Rep. 2021 Jun 16;11:12630. doi: 10.1038/s41598-021-91572-x (PMC8209158; doi:10.1038/s41598-021-91572-x)
Supplement: Supplementary file 1 — Supplementary Information. [file 41598_2021_91572_MOESM1_ESM.pdf]

Supplementary information

Advanced Mycelium Materials as Potential Self-Growing Biomedical Scaffolds

*Maria Elena Antinori[1,2], Marco Contardi[1], Giulia Suarato[1,3], Andrea Armirotti[4], Rosalia Bertorelli[3], Giorgio Mancini[1], Doriana Debellis[5], Athanassia Athanassiou[1]**

Affiliations

[1] Fondazione Istituto Italiano di Tecnologia, Smart Materials, Via Morego 30, 16163 Genova, Italy

[2] DIBRIS, University of Genoa, Genoa, Italy

[3] Fondazione Istituto Italiano di Tecnologia, Translational Pharmacology, Via Morego 30, 16163 Genova, Italy

[4] Fondazione Istituto Italiano di Tecnologia, Analytical Chemistry Lab, Via Morego 30, 16163 Genova, Italy

[5] Fondazione Istituto Italiano di Tecnologia, Electron Microscopy Facility, Via Morego 30, 16163 Genova, Italy

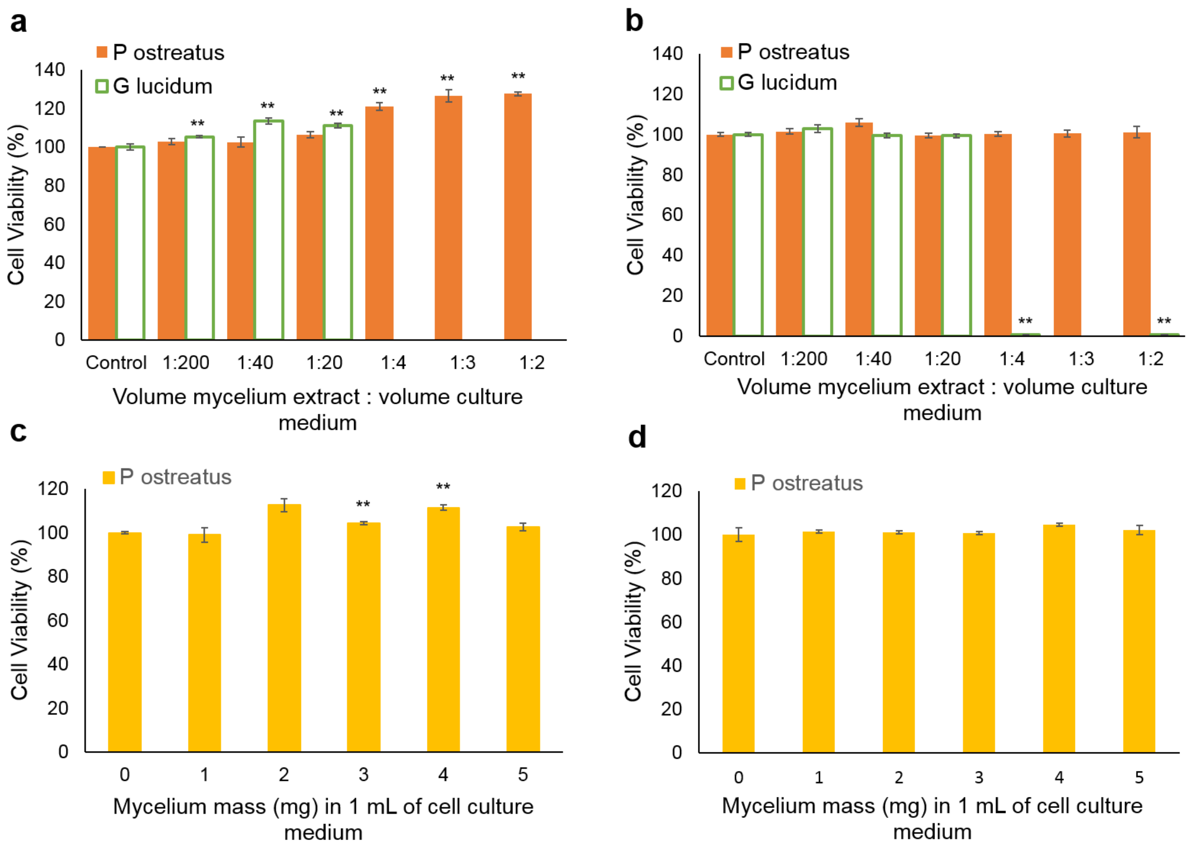

Figure S1. Biocompatibility towards HDFA cells. Primary human fibroblast (HDFa cells) viability in the presence of mycelia extract for 48 hours (a) and 72 hours (b). The reported values indicate the dilutions prepared starting from a stock solution of 20 mg of mycelium in 1 mL of Medium 106 containing growth factors. MTS assay results for the Pleurotus ostreatus semi-contact experiments carried out for 48 hours (c) and 72 hours (d). Data are expressed as average ± standard error and a $p < 0.01$ (**) was considered as significant.

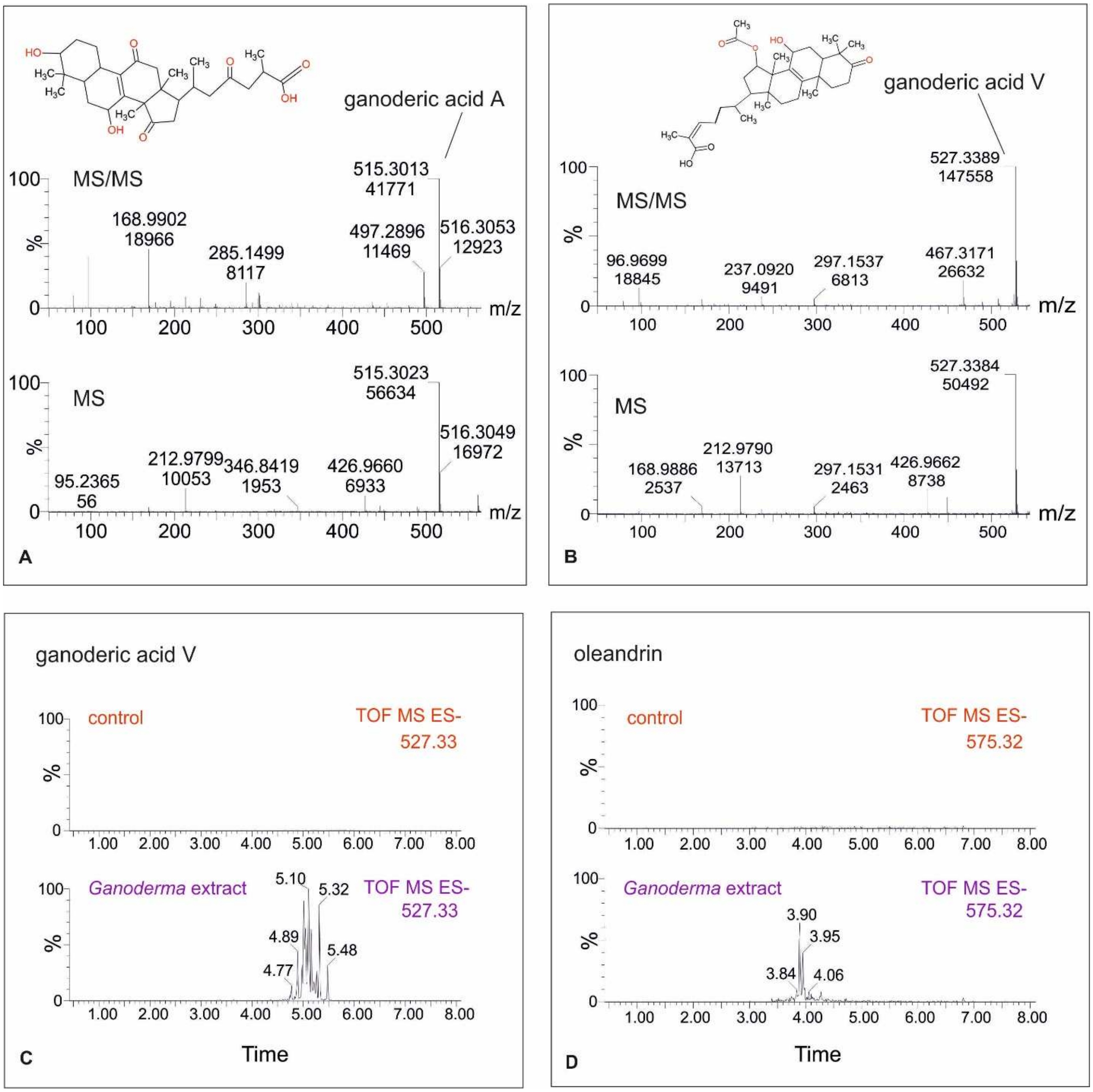

Figure S2. Results of UPLC-MS analysis of extracts and washing PBS from *Ganoderma lucidum*. TOF MS spectra of ganoderic acid A (A), used as standard, and ganoderic acid V (B), effectively detected in the extracts. Traces for extracted ions m/z 527.33 (ganoderic acid V, C) and 575.32 (oleandrin, D).

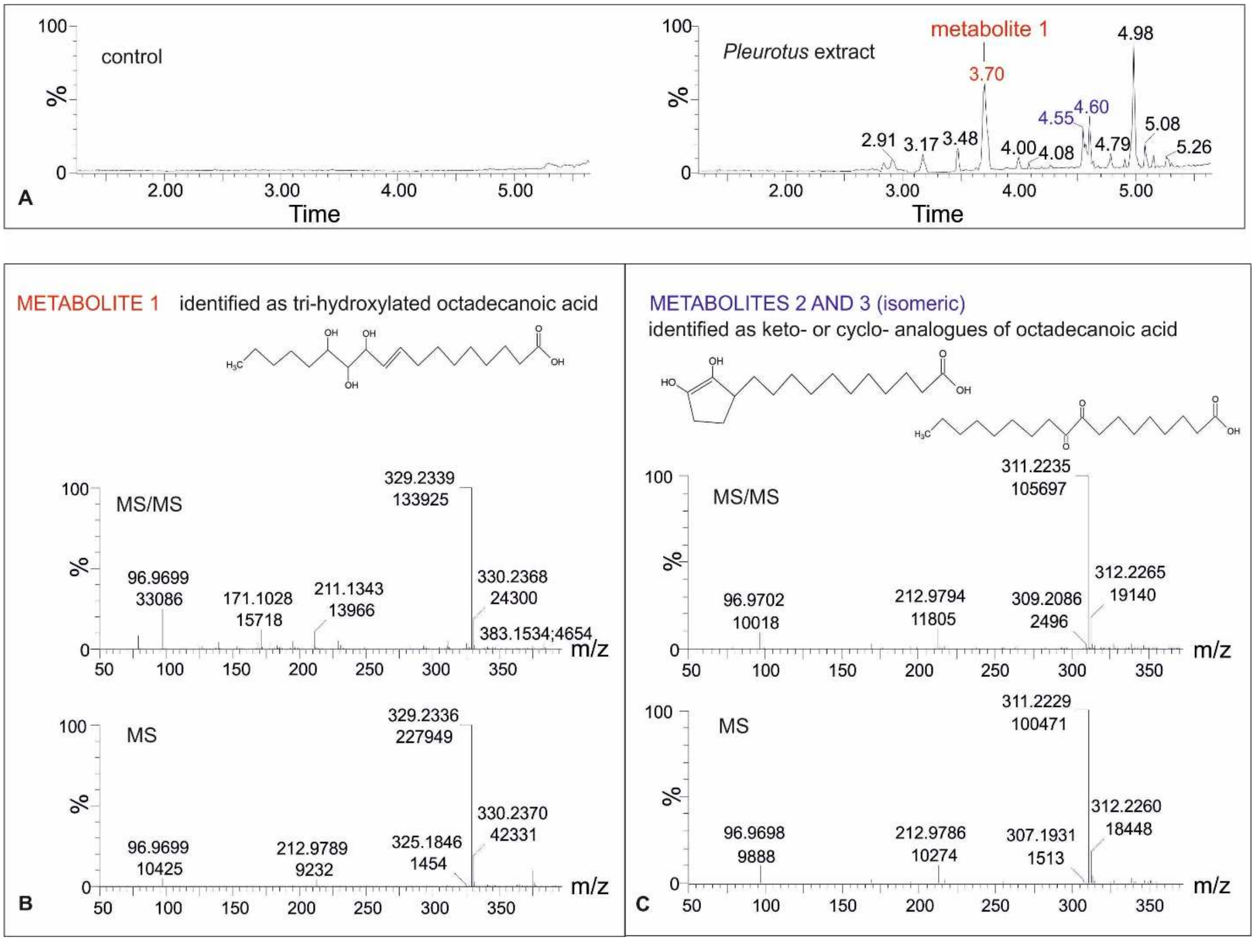

Figure S3. Results of UPLC-MS analysis of extracts and washing PBS from *Pleurotus ostreatus.*

UPLC chromatograms (A) and MS spectra of metabolite 1 (B) and metabolites 2 and 3 (C)
